# Case report: High-grade serous tubo-ovarian carcinoma with *FGFR2::IQCG* fusion and insights into targetability

**DOI:** 10.3389/fonc.2024.1514471

**Published:** 2024-12-20

**Authors:** Reiri Sono, Gottfried E. Konecny, Liying Zhang

**Affiliations:** ^1^ Department of Pathology and Laboratory Medicine, David Geffen School of Medicine, University of California, Los Angeles, Los Angeles, CA, United States; ^2^ Department of Medicine, David Geffen School of Medicine, University of California, Los Angeles, Los Angeles, CA, United States

**Keywords:** case report, high-grade serous tubo-ovarian cancer, *FGFR2*, *FGFR2::IQGC*, novel fusion

## Abstract

*FGFR2* fusion is one of the classes of emerging therapeutic targets of precision oncology and is observed in many solid tumor types. Our understanding of oncogenic mechanisms and therapy effects of molecular targets tends to reflect those occurring in overrepresented tumor types, posing a challenge in therapy planning of the same targets occurring in unusual tumor types. We present a case of a primary high-grade serous tubo-ovarian carcinoma with a novel *FGFR2::IQCG* fusion, an exceedingly rare combination of tumor type and fusion class, with an unusually short-lived response to futibatinib. We review the potential pathogenic mechanism of this fusion and address challenges in predicting targeted therapy efficacy using various assay types and trial designs in heterogeneous tumor types sharing a structural variant.

## 1 Introduction

*FGFR2* fusions are common in a subset of cholangiocarcinoma and lung, breast, thyroid, and prostate adenocarcinoma (1–3) and rare in ovarian adenocarcinomas, while *FGFR2* point mutations or gene amplification is more common (4, 5). To date, there is only one other case report of *FGFR2* fusion-positive ovarian neoplasm (6). The vast majority of *FGFR2* breakpoints result in the truncation of the C-terminal domain of *FGFR2*, which has well-supported *in vitro* evidence to activate FGFR2, seemingly with or without fusing with a dimerization domain provided by its partner (7–13). The Food and Drug Administration (FDA) has approved uses of futibatinib and pemigatinib for *FGFR2* fusion-positive cholangiocarcinoma and an additional use of pemigatinib for myeloid/lymphoid neoplasms with eosinophilia and rearrangement of *PDGFRA/PDGFRB* or *FGFR1* or with *PCM1::JAK2* (14–16). Although their effects vary by cancer type, they are the most effective on intrahepatic cholangiocarcinoma, which is also the most well-represented in this fusion-positive group (17).

We present a case of high-grade serous carcinoma of Mullerian primary with a novel *FGFR2::IQCG* fusion an exceedingly rare combination of tumor type and fusion class, which progressed through multiple lines of therapy before and after a brief period on futibatinib. We illustrate the accompanying molecular findings over time to serve as a starting point in better characterizing the contexts surrounding targetable molecular alterations and affecting therapeutic choices.

## 2 Clinical findings

The patient is a 62-year-old woman initially diagnosed with a primary International Federation of Gynecology and Obstetrics (FIGO) stage IIIC high-grade serous tubo-ovarian carcinoma. The patient had undergone an exploratory laparotomy with total abdominal hysterectomy, bilateral salpingo-oophorectomy, and optimal cytoreductive surgery, followed by six cycles of intraperitoneal and intravenous chemotherapy with cisplatin and paclitaxel. The pre-therapeutic specimen was unavailable for molecular studies. Three years later, the patient presented with elevated CA125 values and a peritoneal recurrence. The molecular panel (FoundationOne CDx) reported that the recurrent tumor sample was homologous recombination deficient (HRD) with 20.3% loss of heterozygosity (LoH; cutoff >16%), microsatellite stable (MSS), and low tumor mutational burden (TMB; 1 mutation/Mb). This was also the first report of an *FGFR2::IQCG* fusion in this patient. Concurrent molecular changes included amplification of *BCL2L1* at chromosome 20q11.21, *C11orf30 (EMSY)* at 11q13.5, *PIK3CA/PRKCI/TERC* at 3q26, and a *TP53* p.F341fs*4 mutation (**Table 1**).

**Table 1:** Summary of FGFR2 fusion breakpoints reported to date in Gao 2018 (2), Hu 2018 (3), and Martignetti 2014 (6), organized by sidedness (5′ or 3′) and cancer type.

FGFR2 breakpoints (| = alternative splice products)	5′								3′						Total
Cancer type	BRCA	CHOL	LIHC	LUSC	OV	STAD	THCA	UCEC	CHOL	LIHC	LUSC	PRAD	THCA	UVM	
Exon 2 last base					1										1
Exon 4 first base														1	1
Exon 4 first base|exon 6 first base|exon 5 first base												2			2
Exon 5 last base								1							1
Exon 8 3rd from last AA; last base on alt transcripts|exon 8 last base	1														1
Exon 9 last base	1	1													2
Exon 12 last base	1														1
Exon 17 last base	2	4	2	2	1	1	1	2							15
Exon 18 first base									2	1	1		1		5
**Total**	5	5	2	2	2	1	1	3	2	1	1	2	1	1	29

Source data are available in **Supplementary Table S3**.

AA, amino acid; alt, alternative; BRCA, breast adenocarcinoma; CHOL, cholangiocarcinoma; LIHC, liver hepatocellular carcinoma; LUSC, lung squamous cell carcinoma; OV, ovarian serous adenocarcinoma; PRAD, prostate adenocarcinoma; STAD, stomach adenocarcinoma; THCA, thyroid carcinoma; UCEC, uterine corpus endometrial carcinoma; UVM, uveal melanoma.

She completed six cycles of second-line chemotherapy with carboplatin and liposomal doxorubicin. One year after its completion, she experienced a second relapse and received a third regimen of six cycles of carboplatin in combination with bevacizumab followed by maintenance therapy with the PARP inhibitor olaparib for 4 months until she experienced a third relapse. Her disease was now deemed platinum-resistant, and two circumscribed lesions were treated with radiation therapy. Six months later, she had a hepatosplenic and pelvic relapse and was enrolled in a clinical trial assessing the folate receptor-targeting antibody–drug conjugate mirvetuximab soravtansine. After treatment of eight cycles over 5 months, she experienced progression of the pelvic disease and was enrolled in another clinical trial assessing the PD-L1 inhibitor atezolizumab in combination with an anti-TIGIT antibody and bevacizumab.

Six months into that clinical trial, her peritoneal disease progressed, and she was subsequently enrolled in a clinical trial assessing futibatinib, a selective, irreversible inhibitor of FGFR 1, 2, 3, and 4. Two cycles (1 month) into the trial, she experienced small bowel obstruction due to interval growths of small bowel serosal implantation. She was taken off the trial and started her seventh regimen of eighth cycles of pemetrexed and bevacizumab. Upon renewed progression, she received an eighth regimen of paclitaxel/gemcitabine and bevacizumab. At that time, a second FoundationOne CDx panel was performed on a new tissue biopsy. HRD was positive again (LoH 25.3%), the microsatellite was stable (MSS), and TMB was low (4 mutations/Mb). *FGFR2::IQCG* fusion and the *TP53* mutation were redemonstrated while previously reported gene amplifications were negative, and two new mutations, *NF1* c.7458-1G>A and *RB1* p.N290fs*11, were detected (**Table 1**).

Following a partial response to the later chemotherapy, imaging demonstrated new progression 3 months after completion of her last chemotherapy. Weekly chemotherapy with topotecan was initiated, which had to be discontinued after 1 month due to rapid progression. The tumor tissue from the second molecular panel had a HER2/neu expression level of +1 on immunohistochemistry, and she received 11 cycles of the HER2-targeting antibody–drug conjugate, fam-trastuzumab deruxtecan-nxki. After this regimen, she was enrolled in a clinical trial assessing the novel second-generation folate receptor-targeting antibody–drug conjugate IMGN151. Following renewed progression, the patient received single-agent weekly paclitaxel but finally opted for palliative/hospice care 9 years after her initial diagnosis of FIGO stage IIIc tubo-ovarian carcinoma.

## 3 Materials and methods

Clinical history, histopathology results, and molecular pathology results were retrieved from the electronic health record system at University of California, Los Angeles.

The tumor tissue tested using FoundationOne^®^ CDx followed the methods detailed in their method validation report (18). Of note, this assay targeted full coding exonic regions and selected intronic regions of *FGFR2* for rearrangement detection.

Non-overlapping curated studies on cBioPortal (https://www.cbioportal.org/) were queried with *FGFR2* as the gene keyword on March 28, 2024. The results were downloaded into a tab-separated text file. All analyses were carried out in Microsoft Excel and are available as **Supplementary Tables**.

## 4 Molecular findings

**Figure 1:**
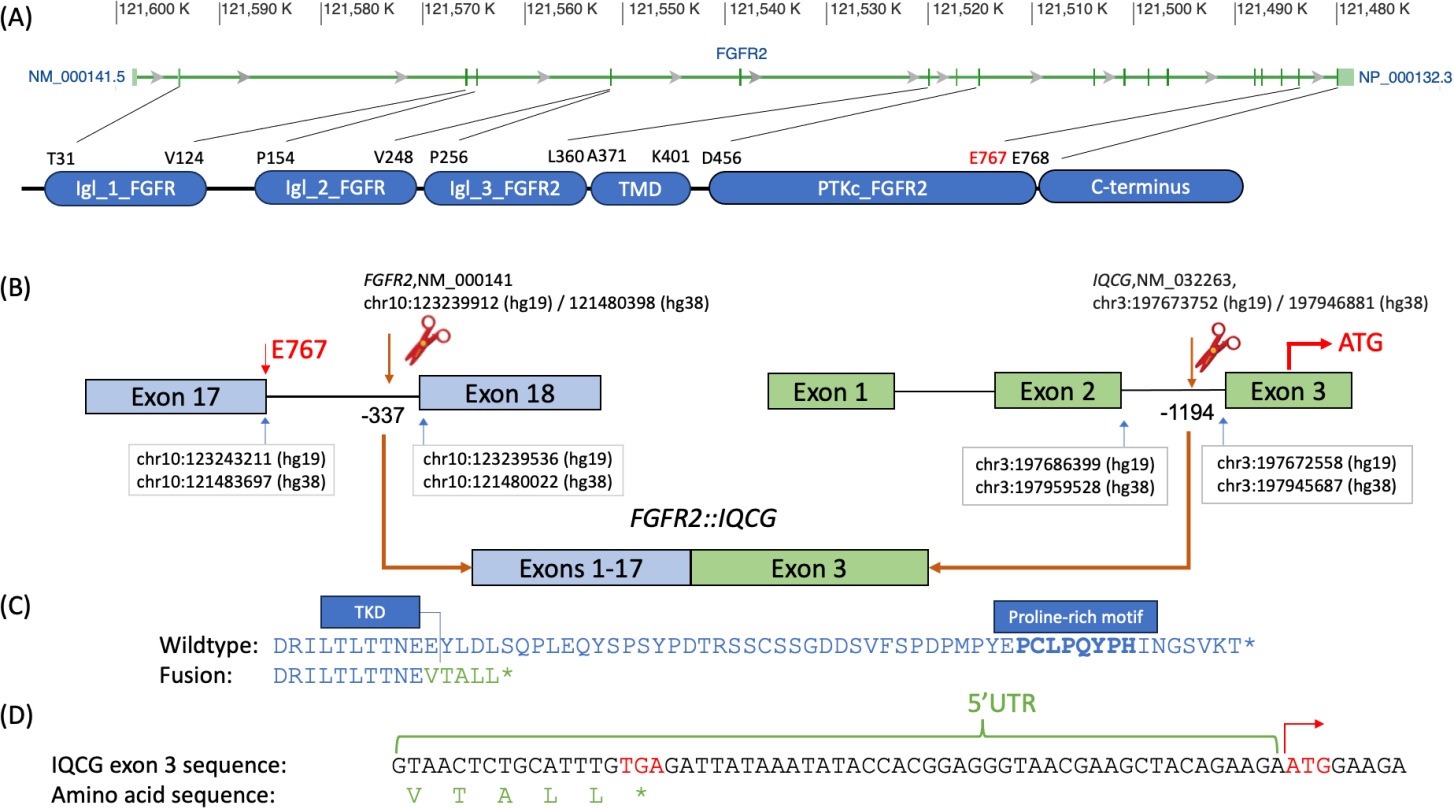
Breakpoint diagram of the tentative *FGFR2::IQCG* fusion protein. **(A)** Exon diagram of *FGFR2* with hg38 chromosomal addresses at the top, excerpted from GenBank (https://www.ncbi.nlm.nih.gov/gene/2263), aligned to a domain diagram of *FGFR2* at the bottom with amino acid abbreviations and positions corresponding to the starts and ends of each domain as defined on GenBank NM_000141. **(B)** Top left, *FGFR2* exons 17 and 18 labeled with the chromosomal addresses of the last base of exon 17 (blue arrow, left), breakpoint base −337 bases from the first base of exon 18 (brown arrow and scissor icon), and first base of exon 18 (blue arrow, right) as well as the 767th amino acid (E767, red) at the 3′ end of exon 17. Top right, *IQCG* exons 1, 2, and 3 labeled with the chromosomal addresses of the last base of exon 2 (blue arrow, left), breakpoint base −1,194 bases from the first base of exon 3 (brown arrow and scissor icon), and first base of exon 3 (blue arrow, right), as well as the opening codon (ATG, red) in the middle of exon 3. Bottom, the proposed fusion product juxtaposing exons 1–17 of *FGFR2* and exon 3 of *IQCG* assuming that all and only the canonical splice sites are used. **(C)** Comparative amino acid sequences of the native *FGFR2* C-terminus (top, blue) and the proposed fusion breakpoint (bottom, blue and green). Top, the DRILTLTTNEE sequence (blue, delimited on the right with a vertical bar) represents the last 11 amino acids of the tyrosine kinase domain (TKD) of *FGFR2*; the boldface PCLPQYPH portion represents the proline-rich motif. Bottom, the VTALL* portion (green) represents the total amino acids contributed by *IQCG*’s 5′UTR readthrough. **(D)** The genomic bases of the whole 5′ UTR of *IQCG* exon 3 up to the opening codon demonstrate that a closing codon of TGA (red) is encountered before the opening ATG on the right (red with a right arrow). Chr, chromosome; IgI_1_FGFR, first immunoglobulin-like domain of fibroblast growth factor receptor; IgI_2_FGFR, second immunoglobulin-like domain of fibroblast growth factor receptor; IgI_3_FGFR2, third immunoglobulin-like domain of fibroblast growth factor receptor 2; PTKc_FGFR2, catalytic domain of the Protein Tyrosine Kinase, Fibroblast Growth Factor Receptor 2; TKD, tyrosine kinase domain; TMD, transmembrane domain; UTR, untranslated region.

*FGFR2::IQCG* is a novel fusion with the *FGFR2* breakpoint at chr10:121480398 (hg38)/10:123239912 (hg19) and *IQCG* at chr3:197946881 (hg38)/3:197673752 (hg19). The *FGFR2* breakpoint is 337 bases upstream of exon 18 of its canonical transcript NM_000141 and preserves all except the last amino acid of the FGFR2 protein tyrosine kinase domain (TKD), which spans AA 456–768 according to GenBank, accessed 11/1/2023 (**Figure 1A**). The *IQCG* breakpoint is 1,194 bases upstream of exon 3, which contains 59 bases in its 5′ untranslated region (5′UTR) (**Figure 1B**). Assuming splicing is not affected, the fusion protein adds five amino acids, VTALL, to the truncated FGFR2 before reaching a stop codon (**Figures 1C, D**).

## 5 Discussion

### 5.1 *FGFR2* fusion-positive neoplasms across anatomical sites

**Figure 2:**
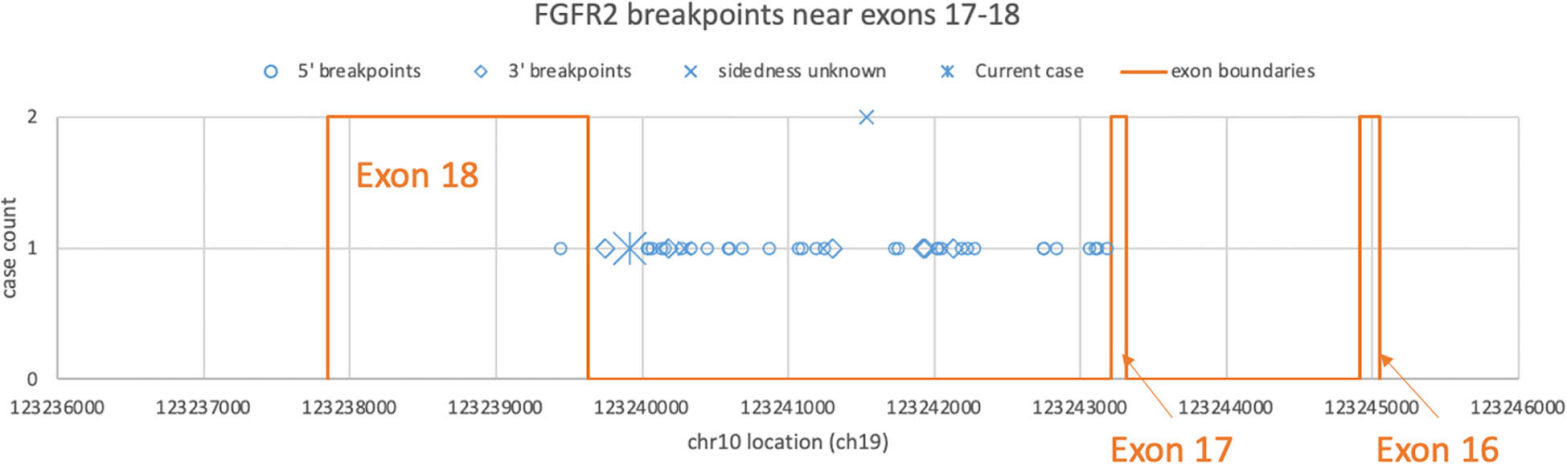
Distribution of FGFR2 breakpoints in genomic DNA drawn from a subset of cBioPortal submissions whose breakpoints fall between exons 16 and 18. Each blue dot represents a submitted case, omitting three outliers each in introns 2, 4, and 8. The star represents the DNA breakpoint of the present case. The orange lines represent the exon boundaries.

Recent high-throughput mRNA sequencing studies (2, 3) and the ovarian neoplasm case (6) spanned 26 samples with *FGFR2* fusions, 21 of which had *FGFR2* as the 5′ partner. Of these 21, 14 had a breakpoint identical to the present case’s prediction (**Table 1**; **Supplementary Table S1**). DNA- or RNA-based assay submissions in cBioPortal listed 123 additional cases with 141 calls, 71 of which reported breakpoint coordinates (**Supplementary Table S2**). Intrahepatic cholangiocarcinoma made up almost one-third of the cases (**Table 2**). There were 33 and eight cases each with 5′ and 3′ *FGFR2* partnership fused with a non-*FGFR2* partner, respectively. One case had RNA evidence of bidirectional transcription of the fusion products. Two additional cases had intragenic fusions or inversions. A total of 55 unique DNA breakpoints within the *FGFR2* coding region were reported, all except six, of which were in intron 17 of the canonical transcript (**Figure 2**; **Supplementary Table S2**).

**Table 2:** Count of unique cBioPortal cases with *FGFR2* fusions by cancer type.

Cancer type	n
Intrahepatic cholangiocarcinoma	55
Cholangiocarcinoma	14
Breast invasive ductal carcinoma	10
Hepatocellular carcinoma plus intrahepatic cholangiocarcinoma	9
Gastric adenocarcinoma	8
Cancer of unknown primary	4
Liver hepatocellular carcinoma	4
Perihilar cholangiocarcinoma	4
Extrahepatic cholangiocarcinoma	3
Hepatocellular carcinoma	3
Lung squamous cell carcinoma	3
Breast invasive carcinoma	2
Lung adenocarcinoma	2
Pancreatic adenocarcinoma	2
Papillary thyroid cancer	2
Bladder urothelial carcinoma	1
Colorectal adenocarcinoma	1
Gallbladder adenocarcinoma	1
Gallbladder cancer	1
Gastric carcinoma other	1
Liver hepatocellular carcinoma plus intrahepatic cholangiocarcinoma	1
Mucinous stomach adenocarcinoma	1
Osteosarcoma	1
Pancreatobiliary ampullary carcinoma	1
Poorly differentiated carcinoma, NOS	1
Poorly differentiated non-small cell lung cancer	1
Serous ovarian cancer	1
Small bowel cancer	1
Stomach adenocarcinoma	1
Synovial sarcoma	1
Uterine endometrioid carcinoma	1
**Total**	141

The numbers include cases with or without a breakpoint coordinate report. Details of each case are available in **Supplementary Table S2**.

One case of ovarian serous adenocarcinoma had a breakpoint at the end of exon 2 with *USP10* as the partner (2, 3), and the other case (6) had an intron 17 breakpoint leading to exon 18 truncation like the present case.

### 5.2 Proposed pathogenic mechanism of *FGFR2* fusions

*FGFR2* alterations disproportionately affect exon 18 at the C-terminus (7–9). The C-terminal changes have multiple modes of activation: loss of the proline-rich Grb2-binding motif, fusion with an external dimerizer, or loss of the more proximal YLDL motif (AA 770-773) by truncation or missense mutations (8, 9).

The proline-rich motif (AA 807–814, boldface in **Figure 1B**) binds Grb2 and keeps FGFR2 at a basal phosphorylation state. Truncation of its last 10 amino acids is sufficient to potentiate constitutive activation (9). This motif also binds and prevents the juxtamembrane domain (JMD; AA 428–441) from binding the FRS2 scaffolding protein (10). The present case, with minimal amino acids contributed by the C-terminal fusion partner, structurally resembles a nonsense variant such as the CT^C3^ construct of Lin et al. (10), which demonstrated enhanced FRS2 binding and ERK phosphorylation.

Cha et al. (11) demonstrated that the Y770 residue was necessary for PLCγ binding and that the L773 residue was necessary for receptor internalization, which agrees with Lin’s CT^C3^ construct showing enhanced plasma membrane localization. Cha also showed that co-mutation led to cooperative transforming activity, which supports the driver potential of the present case with a loss of all domains including and downstream of the YLDL motif.

The need for a dimerizing domain to activate FGFR2 is being debated. Lorenzi et al. (12, 13) showed enhanced FGFR2 activity in an animal cell line transfected with *FGFR2::FRAG1*. The human counterpart of *FRAG1* is identified as *PGAP2* today (19) with no evidence of dimerization. The externally supplied dimerizing domain hypothesis holds for the majority of cases with 3′ partners such as *TACC3* and *BICC1* (20). However, exceptions exist such as intact *FGFR2* placed under a powerful promoter (20) or fusing with 3′ partners with putative but not empirically demonstrated dimerization domain, like *AFF3* (20) or *FAM76A* (6). The *FGFR2::FAM76A* case report (6) is especially relevant since it occurred in a moderately differentiated serous ovarian carcinoma with the same FGFR2 mRNA breakpoint as the present case. The ovarian carcinoma-derived cell line had increased fusion protein expression and proliferation, which was reduced by the pan-FGFR inhibitor BGJ398, now named infigratinib. Thus, *FGFR2* fusions to a non-dimerizing partner may drive tumor growth targetable with an FGFR inhibitor.

### 5.3 Co-occurring potential molecular drivers

At first relapse, there were amplifications of potential driver genes as well as a frameshift truncation variant in *TP53*, suggesting a complex karyotype. Gains in these chromosomal arms are not specific to a molecular subgroup and are all potential drivers (21–23). At the sixth relapse, the amplifications were not detected, while new *NF1* and *RB1* loss-of-function mutations were detected. The contributions of the chromosomal arm gains and point mutations relative to the *FGFR2* fusion cannot be assessed directly. It is noteworthy to highlight that the *FGFR2::IQCG* fusion was identified in tumors from both the initial and sixth relapses, suggesting its potential as a sustained driver of tumor progression, although the unavailability of pre-therapeutic molecular study precludes interpreting whether it played any role in primary chemoresistance.

### 5.4 Limitations of testing methods

The driver potential of a structural variant relative to the intact gene product and relative to all other molecular changes is important for therapy choice but can be interpreted only indirectly. The strand counts of the structural variant versus the intact copy are regularly reported in RNA assays, but they must be compared to the strength of expression in non-tumor cells to assess functional significance. The non-tumor comparator should ideally originate from the same tissue type, but such tissue is not as available as blood or fibroblast. Also, some tumor types have no known normal counterpart. Additionally, the mRNA strand counts do not necessarily translate to protein functionality. Whole-transcriptome analysis is an orthogonal method that provides a higher-level view of the active pathways with utility for tumor classification.

Whether a variant is present in all or a fraction of tumor subclones is another factor affecting therapy effectiveness that routine clinical assays do not report. Subclone resolution is addressable only with single-cell or spatially encoded sequencing. Next-generation sequencing (NGS) of purified nucleic acids, in contrast, affords only an inferred, imprecise subclone-to-mutation mapping under a rare circumstance where the tumor happens to have several morphologically distinct sub-regions whose percent nuclear counts match the variant allele frequencies (VAFs). These limitations in predicting the relative presence of drug targets may explain the limited therapy effect and frequent therapy switching such as that experienced in the present case.

### 5.5 Limitations of FGFR2-targeted therapy

Many 5′ FGFR fusion-positive neoplasms are responsive to FGFR-targeted therapies (1), and the fusion in the present case has ample evidence to support oncogenicity. Many therapy trials of multi-kinase or *FGFR*-targeted inhibitors are broadly recruiting tumor types defined by the fusion (14–16), aiming to study the histology-agnostic therapeutic effect and inevitably skewing the outcomes toward the popular tumor types such as intrahepatic cholangiocarcinoma while blurring the differences between the tumor types clustered as “others”. Futibatinib, an irreversible *FGFR1-4* inhibitor given as the sixth line of therapy to the present case, demonstrated a modest lesion size change in a Phase I dose-expansion study in the 31 cases of “other” tumor types including one case of ovarian carcinoma (17). Objective response rate was higher in cholangiocarcinoma (15.6%, 95% CI 7.8%–26.9%), urothelial cancer (15.8%, 95% CI 3.4%–39.6%), and gastric cancer (22.2%, 95% CI 2.8%–60.0%) cohorts and less than 10% in all others. When stratified by molecular changes, *FGFR2* fusion/rearrangement-positive cohort had the largest target lesion shrinkage, followed by *FGFR2* mutation-, *FGFR3* mutation-, and *FGFR3* fusion/rearrangement-positive cohorts. However, the most responsive two molecular groups largely consist of cholangiocarcinoma, confounding the etiology. Possible reasons for suboptimal responses include co-occurring molecular drivers or tumor microenvironment unique to each cancer type, as well as the *FGFR2* behavior dependent on its fusion partner.

## 6 Conclusion

We present a case of *FGFR2* fusion to a novel 3′ partner at a common breakpoint near the *FGFR2* C-terminus in a high-grade serous Mullerian primary adenocarcinoma, an unusual neoplasm type to harbor this fusion. A review of the literature about its breakpoint and the proposed translation product, which resembles an early truncation of the C-terminal inhibitory domain, supports a likely activation of oncogenic pathways downstream of FGFR2 even in the absence of an extra dimerizer domain provided by the fusion partner. Although activating changes in *FGFR* family receptor tyrosine kinases are known to respond to FGFR inhibitors, the present case terminated her futibatinib trial after two cycles due to small bowel obstruction, which prevented the continuation of this oral medication. The interval growth of serosal implantation is consistent with the limited trial outcomes on ovarian cancer cases. The effects of FGFR inhibitors may be affected by the tumor developmental timeline, subclonality, and precise driving potential of the co-occurring oncogenic drivers such as gain of oncogene-containing chromosomal regions. More cases of similar cancer types harboring *FGFR* fusions will be necessary to shed light on the mechanism of limited efficacy of target therapies.

## Data Availability

The datasets presented in this study can be found in online repositories. The names of the repository/repositories and accession number(s) can be found in the article/**Supplementary Material**.

## Supplementary material

Supplementary Table 1 Curated cases of RNA-based FGFR2 breakpoints from web search.

Supplementary Table 2 Curated cases of DNA-based FGFR2 breakpoints from cBioPortal.
